# Protective Role of Astaxanthin in Regulating Lipopolysaccharide-Induced Inflammation and Apoptosis in Human Neutrophils

**DOI:** 10.3390/cimb46080504

**Published:** 2024-08-05

**Authors:** Seongheon Lee, Sung Kuk Son, Eunye Cho, Sungah Yoo, Eun-A Jang, Sang Hyun Kwak

**Affiliations:** 1 Department of Anesthesiology and Pain Medicine, Chonnam National University Hospital, Chonnam National University Medical School, Gwangju 61469, Republic of Korea; aneshead@gmail.com (S.L.); ths1789@naver.com (S.K.S.); jolivia19@naver.com (E.C.); yoosa1224@naver.com (S.Y.); 2 Department of Anesthesiology and Pain Medicine, Chonnam National University Hospital, School of Dentistry, Chonnam National University, Gwangju 61186, Republic of Korea; namahage@hanmail.net

**Keywords:** astaxanthin, neutrophils, sepsis, inflammation, apoptosis, cytokines, MAPK, NF-κB

## Abstract

Astaxanthin, a keto-carotenoid, is known to have potent antioxidant properties. This study aims to investigate the anti-inflammatory effect of astaxanthin and its mechanism in human neutrophils. The effects of astaxanthin on lipopolysaccharide (LPS)-stimulated human neutrophils were investigated in vitro. Neutrophils were isolated from healthy volunteers and stimulated with LPS in the presence and absence of astaxanthin. We assessed cytokine production, signaling pathway activation via mitogen-activated protein kinases (MAPKs) and nuclear factor kappa B (NF-κB), and apoptosis. Astaxanthin’s impact was evaluated at different concentrations, and both pretreatment and cotreatment protocols were tested. The results demonstrated that astaxanthin significantly reduced the production of pro-inflammatory cytokines TNF-α and IL-1β in LPS-stimulated neutrophils. It effectively inhibited the phosphorylation of ERK1/2 MAPK, without notably affecting p38 MAPK or NF-κB pathways. Furthermore, astaxanthin promoted apoptosis in neutrophils, counteracting the apoptosis-delaying effects of LPS. These effects were more pronounced with pretreatment. In conclusion, astaxanthin has protective effects on inflammatory responses in neutrophils by reducing cytokine production and enhancing apoptosis while selectively modulating intracellular signaling pathways. Astaxanthin demonstrates significant potential as a therapeutic agent in the management of severe inflammatory conditions.

## 1. Introduction

Sepsis remains one of the major challenges in modern medicine, characterized by a complex pathophysiology that often results in multisystem organ failure and a high mortality rate, exceeding 20% among adult patients [1,2,3,4]. This life-threatening condition arises from a dysregulated host response to infection, leading to a deleterious cascade of systemic inflammation, tissue damage, and organ dysfunction.

Neutrophils, the most abundant type of white blood cells in humans, play a critical role in the front-line defense against pathogens through their abilities to execute various cytotoxic mechanisms. Under normal physiologic conditions, neutrophils are maintained in a quiescent state with a short lifespan regulated by apoptosis [5]. This ensures the timely resolution of the inflammatory response without causing excessive harm to host tissue. However, in infectious conditions, neutrophils markedly alter their functional abilities, causing excessive activation with enhanced phagocytic activity and prolonged lifespan.

The overactivation of neutrophils can lead to the uncontrolled release of inflammatory mediators, including tumor necrosis factor-alpha (TNF-α), interleukin-6 (IL-6), and IL-1β. Concurrently, there is the activation of critical intracellular signaling pathways, such as the mitogen-activated protein kinase (MAPK) and nuclear factor kappa B (NF-κB) pathways. The resultant imbalance between pro-inflammatory and anti-inflammatory mechanisms contributes significantly to the pathogenesis of infectious diseases, manifesting as immune dysfunction, tissue inflammation, and subsequent organ damage [6]. This highlights the need for therapeutic strategies aimed at modulating the inflammatory response, particularly through the regulation of cytokine production, signaling pathway activation, and promotion of neutrophil apoptosis in sepsis and other severe infectious conditions. In this context, several studies have attempted to find non-toxic therapeutic agents capable of controlling the inflammatory cascade [7,8,9].

Astaxanthin ((3S,3′S)-3,3′-Dihydroxy-β,β-carotene-4,4′-dione), a keto-carotenoid belonging to the xanthophyll group, is found in several marine organisms, including the freshwater microalgae Haematococcus pluvialis, the yeast fungus Xanthophyllomyces dendrorhous, and species of krill in the Euphausia genus, and contributes to the reddish pigment observed in seafood such as salmon, trout, and shrimp [10,11]. Astaxanthin has gained attention for its potent antioxidant properties by reducing reactive oxygen species, and the scientific evidence supporting its properties and safety led to the United States Food and Drug Administration (FDA) approving astaxanthin as a dietary supplement in 1999 [12,13,14]. Previous studies have also demonstrated that astaxanthin exerts anti-inflammatory effects in various cell types, including macrophages and microglial cells, by modulating MAPK and NF-κB pathway signals. However, the mechanisms and signaling pathways underlying anti-inflammatory effects are not fully understood for human neutrophils.

In the context of clinical application, understanding the effects of astaxanthin in different cell types is crucial because each cell type can have distinct inflammatory pathways and responses. Given the importance of neutrophils in the immune response, we focus specifically on its effects in human neutrophils under lipopolysaccharide (LPS) stimulation. This cell-specific research may help in understanding the comprehensive role of astaxanthin in the immune system and developing tailored therapeutic strategies for severe inflammatory diseases such as sepsis. Therefore, the present study aims to investigate the effects of astaxanthin as both a pretreatment and co-treatment modality on the production of inflammatory cytokines, activation of intracellular signaling pathways, and apoptosis in LPS-stimulated human neutrophils.

## 2. Materials and Methods

### 2.1. Neutrophils Isolation

Neutrophils were isolated from the peripheral blood of volunteers in accordance with the ethical guidelines approved by the institutional review board (IRB no. 2023-154). Written informed consent was obtained from all volunteers. Dextran (6%) was used to precipitate red blood cells (RBCs) by gravity at room temperature for 45 min. Leukocyte-enriched pellets were harvested by centrifugation for 6 min at 1100 rpm and resuspended in platelet-poor plasma. The leukocyte-enriched plasma was then centrifuged with a plasma Percoll gradient (3 mL) for 10 min at 1100 rpm. Neutrophils, located at the 42–51% Percoll layer interface, were collected by centrifugation for 5 min at 3000 rpm after erythrocyte removal with RBC lysis buffer. Finally, the cell suspension was prepared in RPMI 1640 medium supplemented with 10% fetal bovine serum and 1% streptomycin and penicillin (Mediatech, Manassas, VA, USA), ensuring cell viability exceeding 98%, as confirmed by the trypan blue exclusion test.

### 2.2. Cytokine Quantification

**Figure 1:**
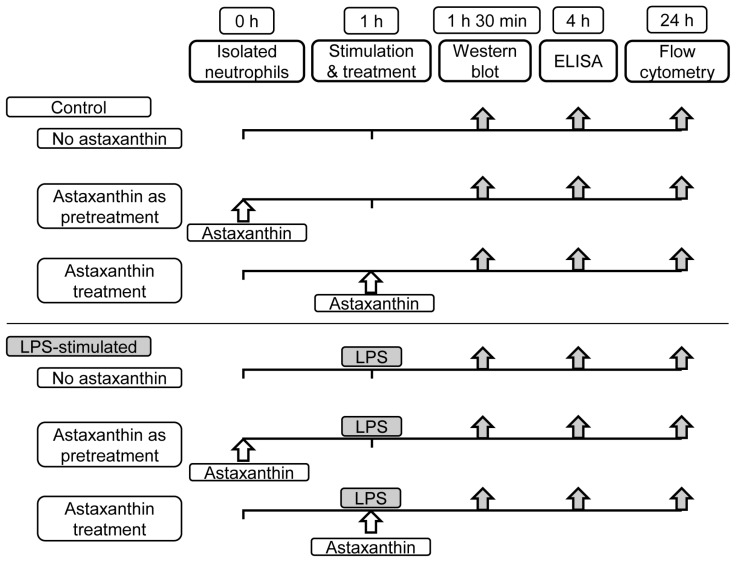
Experimental design used in the study. Astaxanthin (10, 50, 100 μM) was given before or together with LPS 100 ng/mL. Activation of intracellular signaling pathways (p38, ERK1/2, NF-κB) was analyzed by Western blot. Inflammatory cytokine levels were analyzed by ELISA. Quantification of neutrophil apoptosis was analyzed by flow cytometry. LPS, lipopolysaccharide; ERK1/2, extracellular signal-regulated kinase 1/2; NF-κB, nuclear factor kappa B; ELISA, enzyme-linked immunosorbent assay.

To assess the influence of astaxanthin on cytokine production, isolated human neutrophils (5 × 10^6^/mL) were exposed to LPS (100 ng/mL from 055:B5 *Escherichia coli*, Sigma, St. Louis, MO, USA) as an inflammatory stimulus. Astaxanthin (Sigma, St. Louis, MO, USA) was administered in varying concentrations (0, 10, 50, 100 μM) as either pretreatment or cotreatment in 24-well plates for 4 h (Figure 1). The 4 h incubation period was chosen based on preliminary optimization experiments, indicating that this duration allowed for optimal antigen–antibody binding and signal detection in our specific assay setup. Following incubation, levels of inflammatory cytokines (TNF-α, IL-6, and IL-1β) were quantified using enzyme-linked immunosorbent assay (ELISA) kits, according to the manufacturer’s instructions (R and D Systems, Minneapolis, MN, USA). The concentrations of astaxanthin were determined based on previous studies that demonstrated effective anti-inflammatory responses within this range, allowing us to observe dose-dependent effects.

### 2.3. Western Blot Analysis

The modulatory effects of astaxanthin on the phosphorylation status of MAPKs and NF-κB were investigated through Western blot analysis. After exposure to LPS, neutrophils were lysed in protein extraction solution (PRO-PREPTM, Intron biotechnology, Korea). Astaxanthin was administered in varying concentrations as either pretreatment or cotreatment, and the samples were incubated for 30 min and then sonicated for 30 s (Figure 1). Debris from the lysed cells was pelleted by centrifugation for 20 min at 14,000 rpm, and the supernatant was transferred to a new tube and stored at −80 °C until use. Protein concentrations were determined using the bicinchoninic acid (BCA) protein assay kit, according to the manufacturer’s instructions (Pierce Biotechnology, Rockford, IL, USA). The levels of phosphorylated versus total p38 MAPK, ERK1/2 MAPK, and NF-κB (Cell Signaling Technology, Danvers, MA, USA) were assessed by Western blot analysis. The ratio between phosphorylated and total forms was measured using a chemiluminescence system and Image Lab software version 4.1 (Bio-Rad Laboratories, Hercules, CA, USA).

### 2.4. Apoptosis Assessment

The effect of astaxanthin on LPS-induced neutrophil apoptosis was evaluated using fluorescein isothiocyanate annexin V and propidium iodide (FITC-Annexin V/PI), according to the manufacturer’s instructions (BD Biosciences, San Jose, CA, USA), with minor modifications. Neutrophils were cultured with or without LPS for 24 h, with varying concentrations of astaxanthin as either pretreatment or cotreatment (Figure 1). Then, they were washed with phosphate-buffered saline and centrifuged twice for 5 min at 3000 rpm. Cells were incubated with 300 μL of binding buffer containing annexin V/PI for 15–20 min, followed by analysis via flow cytometry within 1 h of labeling to ensure accuracy. Early apoptotic neutrophils were identified by positive FITC-annexin V staining and negative PI staining.

### 2.5. Statistical Analysis

Data were presented as mean ± standard deviation and analyzed for statistical significance using one-way analysis of variance (ANOVA), followed by the Tukey–Kramer multiple comparisons test with SPSS version 21 (IBM Corp., Armonk, NY, USA). A *p*-value of less than 0.05 was considered statistically significant.

## 3. Results

### 3.1. Impact of Astaxanthin on Cytokine Production in LPS-Stimulated Neutrophils

**Figure 2:**
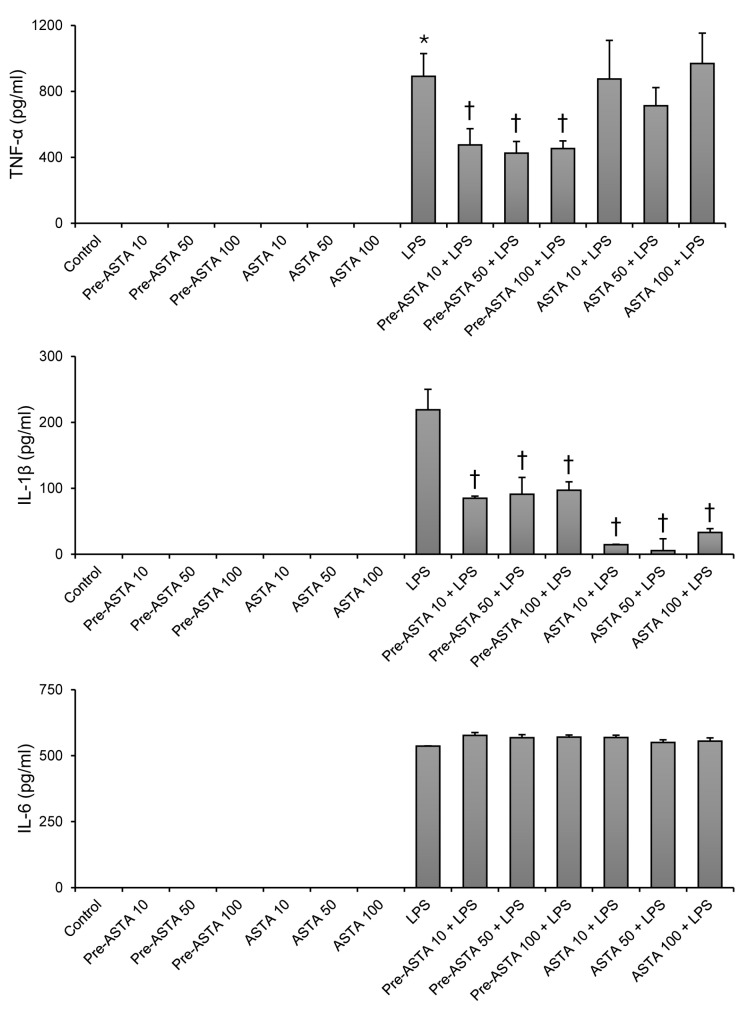
The effects of astaxanthin (ASTA) on inflammatory cytokine (TNF-α, IL-6, IL-1β) expression in human neutrophils stimulated by LPS. After being isolated from human blood, neutrophils were divided into 14 groups, as seen in experimental design (Figure 1). Astaxanthin 10, 50, or 100 μM was added as pretreatment before LPS (pre-ASTA 10, 50, 100 + LPS), or together with LPS as cotreatment (ASTA 10, 50, 100 + LPS). Protein results were determined by ELISA. Data are shown as mean ± SD (n = 4 per group). † *p* < 0.05, vs. LPS. * *p* < 0.05, vs. control. TNF-α, tumor necrosis factor alpha; IL, interleukin; LPS, lipopolysaccharide; ELISA, enzyme-linked immunosorbent assay.

Exposure to LPS significantly increased the production of inflammatory cytokines (TNF-α, IL-6, and IL-1β) in neutrophils, as expected. However, introducing astaxanthin notably altered this response for TNF-α and IL-1β (Figure 2). Specifically, pretreatment with astaxanthin at concentrations of 10, 50, and 100 μM led to a substantial decrease in the level of TNF-α. For instance, TNF-α levels decreased from 892.7 ± 139.0 pg/mL in the LPS-only treated group to 476.9 ± 98.2, 426.3 ± 70.1, and 454.5 ± 46.5 pg/mL, respectively, for each concentration of astaxanthin pretreatment (*p* < 0.05). IL-1β showed a similar trend of reduction across the same astaxanthin concentrations for both pretreatment and cotreatment (*p* < 0.05). LPS increased IL-6 expression, whereas pretreatment or cotreatment with astaxanthin did not significantly alter LPS-induced IL-6 expression (Figure 2).

### 3.2. Effects of Astaxanthin on MAPKs and NF-κB Signaling in LPS-Stimulated Neutrophils

**Figure 3:**
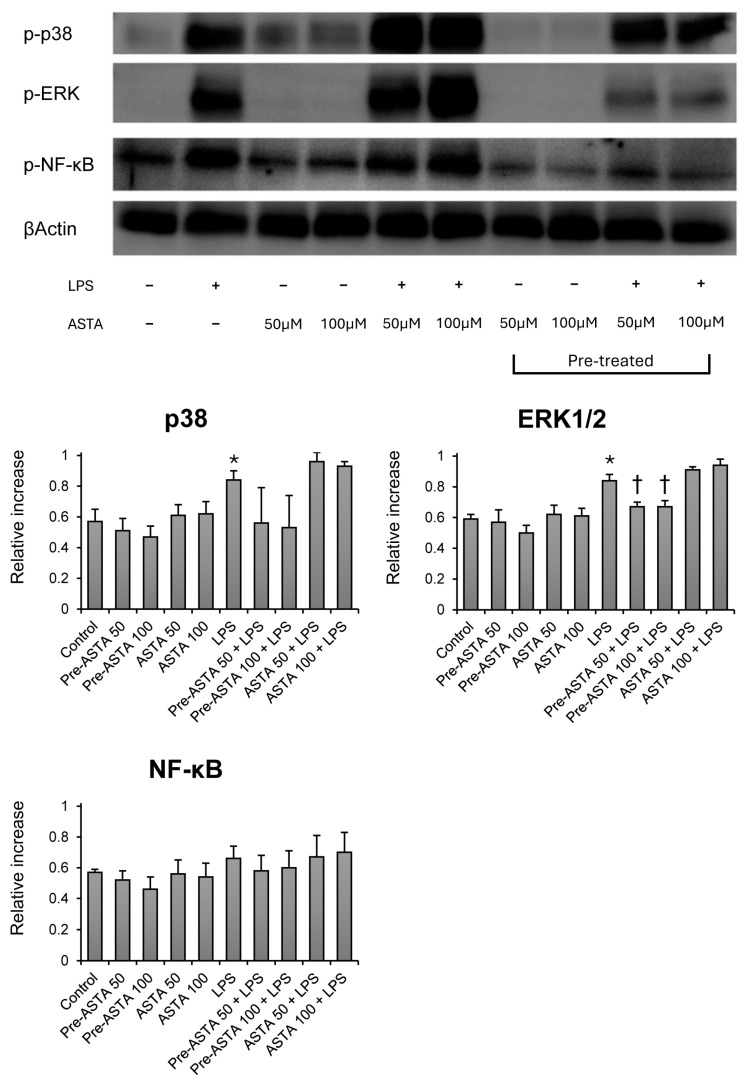
The effects of astaxanthin (ASTA) on mitogen-activated protein kinases (p38 and ERK1/2) and NF-κB activation in human neutrophils stimulated by LPS. Astaxanthin 50 or 100 μM was added as pretreatment before LPS (pre-ASTA 50, 100 + LPS), or together with LPS as cotreatment (ASTA 50, 100 + LPS). The phosphorylation (*p*) levels of p38, ERK1/2, and NF-κB were determined by Western blot analysis. Relative increase is the ratio of phosphorylated to β-actin. Data are shown as mean ± SD (n = 4 per group). † *p* < 0.05, vs. LPS. * *p* < 0.05, vs. control. ERK1/2, extracellular signal-regulated kinase 1/2; NF-κB, nuclear factor kappa B; LPS, lipopolysaccharide.

LPS stimulation activated the phosphorylation of p38 and ERK1/2 MAPKs. Astaxanthin pretreatment significantly reduced the phosphorylation ratio of ERK1/2 compared to the LPS-only group (Figure 3). This effect was not significant for p38 MAPK, indicating that astaxanthin’s modulatory action might be specific to certain signaling molecules. Additionally, our study found no significant change in the activation of NF-κB with astaxanthin treatment, suggesting that its anti-inflammatory effects could be mediated through pathways other than NF-κB in human neutrophils.

### 3.3. Role of Astaxanthin in Modulating Neutrophil Apoptosis Delayed by LPS

**Figure 4:**
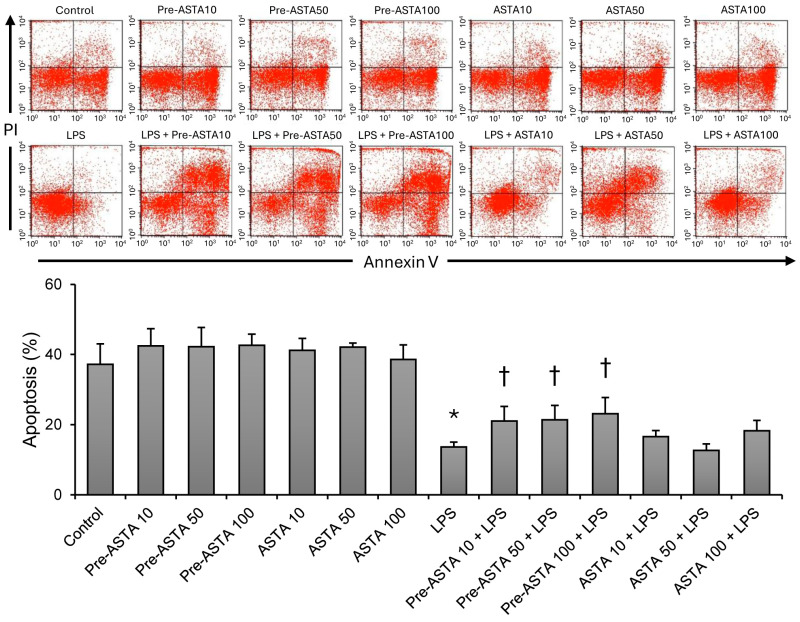
The effects of astaxanthin (ASTA) on apoptosis of human neutrophils stimulated by LPS. Astaxanthin 10, 50, or 100 μM was added as pretreatment before LPS (pre-ASTA 10, 50, 100 + LPS), or together with LPS as cotreatment (ASTA 10, 50, 100 + LPS). Contour diagram of FITC-Annexin V/PI flow cytometry of neutrophils for different groups. The lower right quadrants represent early apoptosis, FITC-Annexin V-positive and PI-negative. One representative experiment out of four is shown. The percentage of neutrophil apoptosis was represented for each group. Data are shown as mean ± SD (n = 4 per group). † *p* < 0.05, vs. LPS. * *p* < 0.05, vs. control. LPS, lipopolysaccharide; ERK1/2, extracellular signal-regulated kinase 1/2; NF-κB, nuclear factor kappa B; ELISA, enzyme-linked immunosorbent assay; FITC-Annexin V/PI, fluorescein isothiocyanate annexin V and propidium iodide.

One of the critical findings of this study is the role of astaxanthin in neutrophil apoptosis. LPS exposure typically delays early apoptosis in neutrophils, contributing to prolonged inflammation. However, astaxanthin pretreatment attenuated this inhibitory effect, promoting early apoptosis even in the presence of LPS (Figure 4). Specifically, neutrophil apoptosis rates increased significantly with astaxanthin pretreatment at all tested concentrations (10, 50, 100 μM) compared to the group treated with LPS alone (13.6 ± 1.4 to 21.0 ± 4.1, 21.4 ± 4.1, 23.1 ± 4.6, respectively; *p* < 0.05).

## 4. Discussion

The primary finding of our investigation reveals the protective role of astaxanthin against the excessive inflammatory responses in human neutrophils, especially when used as a pretreatment. This study affirms astaxanthin’s capacity to selectively modulate key inflammatory pathways, leading to a reduction in cytokine levels and an alteration in cell survival mechanisms. Specifically, astaxanthin significantly diminished the phosphorylation of ERK1/2 MAPK and reduced the levels of TNF-α and IL-1β without notably affecting p38 MAPK and NF-κB pathways. This selective inhibition emphasizes the regulatory effects of astaxanthin on neutrophil activity, which could have profound implications for managing acute inflammatory responses, particularly in conditions like sepsis.

Our findings resonate with and extend previous studies, demonstrating astaxanthin’s efficacy in reducing cytokine expression, improving survival rates, and mitigating organ injury in animal sepsis models [15,16]. The in vitro results presented herein suggest potential mechanisms through which astaxanthin exerts these beneficial effects, proposing the modulation of MAPK signaling pathways as a key factor in its anti-inflammatory action.

The antioxidant properties of astaxanthin have been well documented, along with other promising properties such as anti-cancer, anti-diabetic, neuroprotective, skin-protective, and anti-Helicobacter pylori effects [17,18,19,20,21]. However, its capabilities as an anti-inflammatory agent, particularly in the context of neutrophil-mediated inflammation, are less understood. Our study contributes to filling this knowledge gap by demonstrating astaxanthin’s role in reducing the inflammatory response through ERK1/2 pathway modulation and promoting apoptosis in LPS-stimulated human neutrophils.

In various experimental models, astaxanthin has been shown to prevent the activation of both MAPKs and NF-κB [22,23]. However, its effects may differ significantly across different cell types involved in inflammation. While astaxanthin reduced the phosphorylation of NF-κB in macrophages [23], in the present study, it effectively reduced the phosphorylation of ERK1/2 MAPK but did not affect NF-κB in human neutrophils. The ERK1/2 MAPK pathway is known to regulate pro-inflammatory cytokine production and cell survival. These findings suggest that astaxanthin may modulate the NF-κB pathway differently depending on the cell type. In neutrophils, astaxanthin appears to have a more pronounced effect on the ERK1/2 MAPK pathway, indicating a cell-type-specific mechanism of action. Additionally, the NF-κB pathway might be less sensitive to the concentrations of astaxanthin used in this study or might require different timing of treatment to observe significant effects.

In a study by Macedo et al. [24], astaxanthin was shown to significantly reduce IL-6 and TNF-α production in LPS-stimulated neutrophils, highlighting its potent anti-inflammatory properties. Our findings align with their results regarding the reduction of TNF-α; however, we observed no significant effect on IL-6 levels. This difference could stem from variations in experimental conditions, such as astaxanthin concentration, exposure duration, or specific methodological approaches. Macedo et al. used a concentration of 5 μM astaxanthin with an 18 h incubation period, whereas our study employed different concentrations and incubation times. Further research is needed to reconcile these differences and fully elucidate the mechanisms through which astaxanthin modulates cytokine production in neutrophils.

Consistent with previous findings, our study observed that LPS stimulation decreased neutrophil apoptosis. Notably, this effect was attenuated by astaxanthin, which suggests that astaxanthin not only modulates the inflammatory response at the molecular level but also influences the lifespan of neutrophils under inflammatory conditions. Astaxanthin’s ability to enhance apoptosis in LPS-stimulated neutrophils may be attributed to its inhibition of the ERK1/2 MAPK pathway. The ERK1/2 MAPK pathway is crucial for cell survival and proliferation, and its inhibition can promote apoptosis by downregulating survival signals and upregulating pro-apoptotic factors. By diminishing the phosphorylation of ERK1/2, astaxanthin reduces the survival signals, thereby enhancing apoptosis.

Furthermore, our results indicate that the protective effects of astaxanthin might be more pronounced when administered as a pretreatment rather than a cotreatment in LPS-stimulated human neutrophils. This suggests that the timing of astaxanthin administration is critical for maximizing its therapeutic benefits.

This study has several limitations, including its in vitro nature and the need for in vivo validation. Additionally, while our study showed that pretreatment with astaxanthin is highly effective, it is often impractical to administer a preventive treatment for inflammatory diseases in clinical settings. Therefore, further research should explore the efficacy of astaxanthin when administered at the onset or after the onset of inflammation. Furthermore, we did not conduct toxicity studies on non-immune cells. However, the existing literature extensively documents the safety profile of astaxanthin, particularly noting its FDA approval for use as a dietary supplement and food additive. Several studies have demonstrated the low toxicity of astaxanthin in various non-immune cell types, supporting its safety for broader applications.

In conclusion, astaxanthin significantly diminished the phosphorylation of ERK1/2 MAPK, reduced the levels of TNF-α and IL-1β, and enhanced apoptosis in human neutrophils. These finding highlight astaxanthin’s efficacy in inhibiting the ERK1/2 MAPK pathway associated with neutrophil activation, suggesting astaxanthin’s potential as a treatment option for severe inflammatory conditions.

## Data Availability

The original contributions presented in the study are included in the article; further inquiries can be directed to the corresponding author.
